# Association of *CTLA-4* and *IL-4* polymorphisms in viral induced liver cancer

**DOI:** 10.1186/s12885-022-09633-x

**Published:** 2022-05-07

**Authors:** Maria Shabbir, Yasmin Badshah, Khushbukhat Khan, Janeen H. Trembley, Areeb Rizwan, Fatima Faraz, Syeda Alveena Shah, Mahrukh Farooqi, Naeem Mahmood Ashraf, Tayyaba Afsar, Ali Almajwal, Nawaf W. Alruwaili, Suhail Razak

**Affiliations:** 1 Department of Healthcare Biotechnology, Atta-ur-Rahman School of Applied Biosciences (ASAB), National University of Sciences and Technology (NUST), Islamabad, Pakistan; 2 Department of Biochemistry, University of Gujrat, Gujrat, Pakistan; 3 Minneapolis VA Health Care System Research Service, Minneapolis, MN USA; 4 Department of Laboratory Medicine and Pathology, University of Minnesota, Minneapolis, MN USA; 5 Masonic Cancer Center, University of Minnesota, Minneapolis, MN USA; 6 Department of Community Health Sciences, College of Applied Medical Sciences, King Saud University, Riyadh, Saudi Arabia

**Keywords:** HCC, *CTLA-4*, Gene polymorphism, *IL-4*, HCV

## Abstract

**Background:**

Hepatocellular carcinoma (HCC) is one of the most prevalent types of cancer and is responsible for close to one million annual deaths globally. In Pakistan, HCC accounts for 10.7% of cancer incidence. Prior studies indicated an association between interleukin 4 (*IL-4*) and cytotoxic T lymphocyte protein 4 (*CTLA-4*) gene polymorphisms in many types of cancers, including HCC that are either hepatitis B virus (HBV)- or hepatitis C Virus (HCV)-induced. The association of *IL-4* and *CTLA-4* genetic polymorphisms with HCV-induced HCC is not yet determined in the Pakistani population. Therefore, this research is designed to investigate the implication of *IL-4* and *CTLA-4* gene polymorphisms by determining the association of *IL-4* -590 C/T (rs2243250) and *CTLA-4* + 49 A/G (rs231775) with HCC in Pakistan.

**Methods:**

Different bioinformatics tools were employed to determine the pathogenicity of these polymorphisms. Samples were collected from HCV-induced HCC patients, followed by DNA extraction and ARMS-PCR analysis.

**Results:**

The SNP analysis results indicated a positive association of *IL-4* -590C/T and *CTLA-4* + 49A/G gene polymorphisms with HCV-induced HCC in Pakistan. The *CTLA-4* polymorphism might enhance therapeutic efficiency of HCC chemotherapy medicines. The *IL-4* polymorphism might introduce new transcription factor binding site in *IL-4* promoter region.

**Conclusion:**

This study delineated risk factor alleles in *CTLA-4* and *IL-4* genes associated with HCV-mediated HCC among Pakistani patients that may have application to serve as genetic markers for pre- and early diagnosis and prognosis of HCC in HCV patients.

## Background

Hepatocellular carcinoma (HCC) is a multifactorial primary liver malignancy caused by excessive alcohol intake, obesity and viral infections [1]. Globally, hepatitis B and C virus infections are a major cause of HCC. Chronic HBV and HCV infections have been responsible for 44% and 22% of HCC cases, respectively [2]. HCC is responsible for almost 830,180 deaths around the globe [3]. In Pakistan, about 70% of HCC is imputed to HCV, whereas HBV is the major etiological contributor of HCC disease in Asian and Pacific countries [4].

HCV promotes generation of cytokines which contribute to HCC progression. Cancer-associated genetic polymorphisms have been identified in cytokines that might facilitate their targeting for anti-cancer therapies [5]. Cytokine *IL-4* is involved in regulation of various important biological functions including B-cell mitogenesis, class switching to IgE, cell homeostasis, and tissue repair [6]. During T-cell signalling, *IL-4* promotes T-cell differentiation. In cancer, *IL-4* functions in the tumor microenvironment to mediate pro-tumor activity by activating tumor-associated or myeloid-derived suppressor cell (MDSC) associated macrophages [7]. Compared to *IL-4*, *CTLA-4CTLA-4* acts as a negative regulator of T-cell signalling. It is present on T-cells and binds B7-1 (CD-80) and B7-2 ligands (CD-86). It induces changes that directly stop TCR immune synapse and block CD28 signaling, thus downregulating interaction of T-cells with antigen-presenting cells [8]. Antibodies that target *CTLA-4CTLA-4* are used clinically as anti-cancer immunotherapeutic agents [9].

Genetic variants of *IL-4* and *CTLA-4* are reported to have association with several cancers including HCC, colorectal cancer, and head and neck cancer [10–14]. A recent publication indicated an *IL-4IL-4* variant (rs2243250) with relation to HCV-induced HCC in an Egyptian population [15]. Previously, its association was delineated in a Chinese population [16]. Similarly, association between *CTLA-4* rs231775 and HCV-induced HCC was found in the Chinese Han population [17] and an Egyptian population [18]. Previous studies have delineated the contribution of pathogenic single nucleotide polymorphisms (SNPs) on clinical outcomes in cancer patients. The presence of certain SNPs boosts the efficiency of some treatment drug’s efficacy, leading to increased patient survival rate. For example, the genetic variant rs9582036 was identified in the VEGFR1 receptor that increased patient survival after treatment with bevacizumab [19]. Likewise, HCC patients having the KDR gene rs1870377 AA genotype were reported to show better response to first-line therapy sorafenib [19].

In cancer, *CTLA-4* mediated modulation of *IL-4IL-4* is responsible for immune dysregulation [20]. During HBV infection, up-regulated expression of *CTLA-4* results in the increased activity of *IL-4* that functions to generate anti-infection response [21]. During HIV infection, *CTLA-4* and *IL-4* polymorphism influenced the treatment response in patients [22]. Similarly, *CTLA-4* and *IL-4* polymorphism is also reported to induce HCC progression [23]. The association of genetic polymorphisms of *IL-4* and *CTLA-4* with HCV-mediated HCC is not investigated in the Pakistani population. The objectives of the current research include the prediction of pathogenicity of *IL-4* -590 C/T (rs2243250) and *CTLA-4* + 49 A/G (rs231775) and the study of association between *IL-4* -590 C/T (rs2243250) and *CTLA-4* + 49 A/G (rs231775) polymorphisms with HCV-induced HCC. The *IL-4* polymorphism lies in the promoter region; therefore, the potential influence of *IL-4* -590 C/T polymorphism on the regulation of *IL-4* expression was also estimated. Structural change in *CTLA-4* protein due to the polymorphism was predicted and investigation for *CTLA-4* structural changes on the therapeutic efficiency of HCC first and second line therapeutics was carried out. This study determined putative pre-diagnostic genetic marker for HCV-induced HCC and provided insight on functional impact of *IL-4* and *CTLA-4* genetic variants in HCV-mediated HCC.

## Methods

### In silico method

#### Data retrieval

SNPs of *IL-4* and *CTLA-4* were selected by identifying their integral role in several diseases through literature review [15, 24, 25]. Information regarding the variants of *IL-4* and *CTLA-4* was retrieved from ENSEMBL having genome assembly “GRCh38:CM000667.2” & “GRCh38:CM000664.2”, respectively. ENSEMBL and RegulomeDB were employed to get co-ordinates of *IL-4* (+ 49 A/G) and *CTLA-4* (-590 C/T) SNP and mapped on genome assembly GRCH38.p13. Protein data bank was accessed for downloading protein structures of *CTLA-4* and *IL-4*.

#### SNP Analysis

Pathogenicity of *IL-4* SNP (Variant ID: rs2243250) and *CTLA-4* SNP (ID: rs231775) was predicted using the following six tools: SIFT, PolyPhen2.0, REVEL, MetaLR, MutationAssessor and CADD [26]. SIFT and PolyPhen2.0 predicts protein function changes based on homology in the primary sequence and physiochemical relationship between neighboring amino acids. REVEL integrates prediction scores of SIFT, PolyPhen, MutationAssessor, MetaLR, REVEL, and CADD to make a pathogenicity prediction for a variant. MetaLR uses allele frequency and logistic regression integrating variant deleteriousness scores to predict impact of missense variants. MutationAssessor employs evolutionary conservation knowledge for predicting functionality alteration of the protein on the basis of homology. CADD is used for the prediction of SNP variants deleteriousness via functional or evolutionary conservation data. Regulome DB was used to determine the coordinates, rank, and score of the SNPs [27].

#### Transcription regulation prediction

Alibaba 2.0 was employed to predict the alteration in transcription factor binding sites (www.gene-regulation.com). HOPE project analysis was used to determine protein structural alteration and predict resultant pathogenicity (www.cmbi.umcn.nl/hope/).

#### In situ mutagenesis

Protein tertiary structure for *CTLA-4* was retrieved from Alphafold database (AF-P16410-F1). Mutation in wildtype *CTLA-4* structure was introduced through the mutagenesis tool of PyMol v4.0.2. Both mutated and wildtype structures of *CTLA-4* were superimposed to highlight differences.

#### Molecular docking

Chemical structures for Avastin (PubChem 349,985,080) and Sorafinib (PubChem 216,239) were downloaded from PubChem database. Docking of the drugs against *CTLA-4* (AF-P16410-F1) was done in CB Dock and the vina scores and cavity sizes were retrieved. PyMOL was used for docked structure visualization (http://clab.labshare.cn/cb-dock/php/). LigPlot (version v.1.4.5) was used to visualize hydrogen bonds and hydrophobic interactions between protein and ligand [28].

### In vitro method

#### Study design and patient selection

A retrospective case–control study was conducted in which patients were recruited from the Combined Military Hospital Rawalpindi, Pakistan. The sample size (N) was 429, out of which 213 were HCC patients and 216 were control patients. The inclusion criterion was set as HCV-infected HCC patients and all other HCC patients were excluded. The confirmation of HCV infection in patients was achieved by quantifying the viral load through quantitative-reverse transcriptase polymerase chain reaction (qRT-PCR). Isolation of HCV RNA from the patients’ plasma was performed through Instant virus RNA kit CE IVD ªAnalytik Jena. TaqMan probes were employed for quantifying the HCV in iQ5 real-time PCR detection system ªBio-Rad Laboratories. Healthy subjects were used as control. Sample size was estimated through the procedure explained by Suresh and Chandrashekara (2015) [29] and validated using the G*Power Software version 3.1.9.2 for Windows. Sample collection was performed after written consent was obtained from the subjects. The study was approved by the Institutional Review Board committee, National University of Sciences and Technology, Islamabad, Pakistan (IRB No. 04–2019-03/06). The study was performed in accordance to the principles of the Declaration of Helsinki [30].

#### Design of primers

Primer1 (http://primer1.soton.ac.uk/primer1.html) was used for designing the allele-specific primer sets for *IL4* -590 C/T (rs2243250) and *CTLA4* + 49 A/G genes. For *CTLA4*, forward and reverse primer sequences used as internal controls were CACAAGGCTCAGCTGAACCTGGATG (for Allele A) and ACAGGAGAGTGCAGGGCCAGGTCCTAGT (for Allele B), respectively. Forward and reverse outer primers were GTGGGTTCAAACACATTTCAAAGCTTCAGG and TCCATCTTCATGCTCCAAAAGTCTCACTC, respectively. For *IL4*, forward and reverse primer sequences used as internal controls were TCACGGATTTCTGTTGTGTTTC and GCCTCCCAACCATTCCCTTA, respectively. While ACACTAAACTTGGGAGAACATTGTC for detection of C allele and ACACTAAACTTGGGAGAACATTGTT for T allele detection for used.

#### DNA extraction

Total DNA was isolated from blood using standardized phenol–chloroform protocol [31].

#### Tetra amplification refractory mutation system polymerase chain reaction (Tetra ARMS-PCR)

Tetra ARMS-PCR was used for identification of *CTLA-4* + 49 A/G and *IL-4* -590 C/T polymorphism in genes. Reaction mixture (total volume 20 µl) was prepared by adding 5X buffer solution (4μL), 2 mM dNTPs (2μL), Taq polymerase (0.5 U), primer (10 pmol each), distilled water (10.9 μL) and DNA template (1 μL). The conditions used in PCR were: Denaturation (95˚C for 4 min), 25 cycles of denaturation (95 °C for 45 s), annealing (58 °C for 45 s), and extension (72 °C for 45 s), and a final extension (72 °C for 5 min). Two percent agarose gel was used to analyze the PCR product of ARMS-PCR. A 100 bp ladder was also loaded alongside the PCR products for comparison of size. The results were analyzed by Wealtec dolphin-doc gel analysis systems.

### Statistical analysis

Microsoft office 2016 Excel (Rehmond, WA, USA) was used for organization of the genotyping results. Statistical significance of results was tested using GraphPad Prism software ver8.0.1 (GraphPad Software Inc., San Diego, CA, USA). Genotype distribution of gene polymorphism and their associated strength (odds ratio, OR and relative risk, RR) in patients and healthy control was calculated by two-way Fisher’s exact test. Koopman asymptotic score and Baptista-Pike method was computed for the calculation of OR and RR, respectively. A probability of less than 0.05 was taken as significant.

## Results

### In silico prediction of pathogenicity for *CTLA-4* SNP + 49A/G

RegulomeDB provided the coordinates, score, and rank of the variant (Variant ID: rs231775). Rank of *CTLA-4* + 49A/G polymorphism was 5, depicting transcription factor binding sites and DNase peak in *CTLA-4*. The score of this SNP was 0.13454, showing less regulatory functional role of the SNP. ENSEMBLE indicated *CTLA-4* + 49A/G SNP as a missense variant. Table 1 includes RegulomeDB annotation score and information regarding the chromosomal location (Chr:bp), allelic mutation (A), amino acid alteration (AA) and coordinates (AA coord.) of *CTLA-4* SNP. In silico pathogenicity prediction results obtained for CTLA + 49A/G polymorphism (Variant ID: rs231775) were: SIFT “Tolerated” (score: 0.2), PolyPhen “Benign” (score: 0.009), CADD “Likely Benign” (Score: 0), REVEL “Likely Benign” (Score: 0.007), MetaLR “Tolerated” (Score: 0) and MutationAssessor “low” (Score: 0.28). Overall, *CTLA-4* SNP rs231775 was predicted as “benign or non-pathogenic”**.**

**Table 1:** Annotation scores and chromosomal location (Chr: bp), allelic mutation (A), amino acid alteration (AA) and coordinates (AA coord.) of CTLA4 and IL4 SNP

		**CTLA4**	**IL4**
**ENSEMBLE**	**Variant ID**	rs231775	rs2243250
	**Chr:bp**	2:203,867,991	5:132,009,153
	**Alleles**	A/G	C/T
	**Variant type**	Missense	Promoter region
	**AA**	T/A	–
	**AA coord**	17	–
**Regulome Annotation**	**SCORE**	0.13454	0.60906
	**RANK**	5	4

### HOPE structural alteration analysis for *CTLA-4*

HOPE analysis was employed to predict the potential influence of this SNP on protein structure and function. HOPE predicted that *CTLA-4* SNP (Variant ID: rs231775) mutation (threonine converted to alanine) leads to substitution of a small sized amino acid residue that is more hydrophobic than the wild type residue. The substitution of alanine may affect the interaction of *CTLA-4* with other proteins, and increased hydrophobicity may alter folding of the protein due to loss of H-bonds. The altered amino acid was not located in the conserved region, suggesting that this mutation may not be highly damaging.

### *CTLA-4* variant influence on therapeutic efficiency of chemotherapeutic drugs

**Fig. 1:**
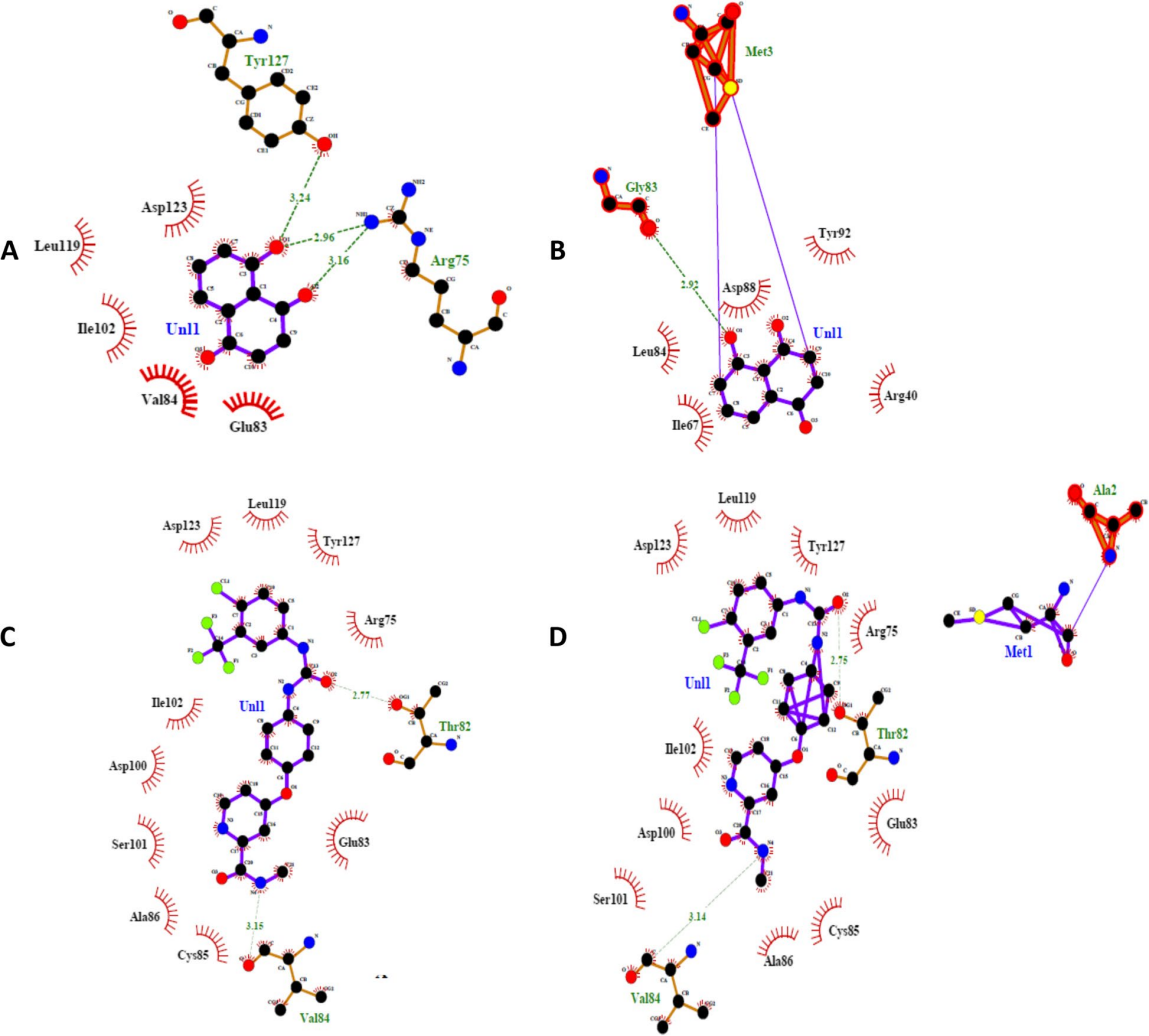
Molecular interaction between *CTLA-4* wildtype/mutated and Avastin and Sorafenib predicted through LigPlot. **A** *CTLA-4* (wildtype) and Avastin complex. **B** *CTLA-4* (mutated) and Avastin complex. **C** *CTLA-4* (wildtype) and Sorafebin complex and. **D** *CTLA-4* (mutated) and Sorafebin complex. Purple lines represent interaction between ligand atoms. Orange line depicts interaction between protein atoms. Hydrogen bonding is shown in green dotted lines. Red spiked semi circles denote hydrophobic interactions. Purple line between ligand and amino acid represents covalent bonds

FDA approved Sorafenib is a first line treatment drug, whereas Avastin is a second line treatment drug for HCC. Both drugs were docked through CB-Dock with wildtype and mutated *CTLA-4* protein to analyse the influence of SNP on binding energy and interaction of *CTLA-4* with Avastin and Sorafenib. Table 2 depicts results obtained via CB-Dock with the Vina score and Cavity size of different docked structures. Molecular docking outcomes indicated that introduction of alanine in place of threonine at position 17 in *CTLA-4* resulted in the addition of a covalent bond in both *CTLA-4*-Avastin and *CTLA-4*-Sorfanib complexes. Avastin binding with *CTLA-4* was possible due to generation of five hydrophobic and two hydrogen bond interactions. Amino acids Glu83, Val84, Ile102, Leu119, and Asp123 participated in non-electrostatic interactions, whereas amino acids Arg75 and Tyr127 hydrogen bonded with Avastin. Residue Arg75 made two hydrogen bonds with the oxygen atoms of Avastin with estimated distance 2.96 Å and 3.19 Å (Fig. 1a). *CTLA-4* + 49A/G polymorphism reduced the number of hydrogen bonds and introduced a covalent bond (Met3) (Fig. 1b). Similar influence of *CTLA-4* polymorphism was found for the *CTLA-4* and Sorafenib model complex. Sorafenib interacted with *CTLA-4* by making two hydrogen bonds (Thr82 and Val84) and ten hydrophobic interactions (Cys85, Glu83, Ala86, Ser101, Asp100, Ile102, Asp123, Leu119, Tyr127 and Arg75). Allele G substitution in *CTLA-4* resulted in the addition of four hydrophobic interactions along with a covalent bond (Fig. 1c and 1d).

**Table 2:** CB Dock scores as a result of docking between CTLA4/IL4 (Proteins) & Tetrahydroxyflavanone (ligand)

Protein	Drug	Vina score
CTLA4 (wild)	Avastin	-5.1
	Sorafinib	-7.3
CTLA4 (Mutated)	Avastin	-5.0
	Sorafinib	-7.3

### In silico prediction of SNP pathogenic impact on *IL-4*

RegulomeDB ranked the *IL-4* SNP at 4, indicating transcription factor binding sites and DNase peak, and a score of 0.60906 suggested high regulatory function of the SNP (Table 1). Alibaba 2.0 transcription site analysis estimated the generation of two additional transcription binding sites for MIG1 and SP1 due to *IL-4* rs2243250. SP1 is located within the E1 region that is responsible for enhancer activity. This SP1 site-generating mutation is thought to have no effect on normal functioning of the E1 region, but more research is needed to draw any conclusion [32].

### Viral load of HCV in HCV-induced HCC patients

Viral load of HCV was determined through qRT-PCR analysis in the plasma of HCV-induced HCC patients to ensure the HCV infection. Viral load more than 800,000 IU/L is considered high. In this study, high viral load of HCV was observed and mean viral load along with standard deviation found was 339,840,893.2 ± 1,092,261,683 IU/L.

### Comparison of *CTLA-4* + 49A/G* and IL-4* -590 C/T genotypes in HCV-associated HCC patients

Association of *CTLA-4* and *IL-4* polymorphism with pathogenicity of HCV-induced HCC was evaluated through ARMS-PCR in 213 patient samples. Outcomes indicated that the prevalence of genotype AG (62.9%) in *CTLA-4* was more than GG genotype (37.1%) (Table 3). The OR and RR scores indicated that individuals with AG genotype are more at risk of developing HCV-induced HCC (< 0.0001). *IL-4* genotypes CC and CT had association with HCV-induced HCC (< 0.0001). However, values obtained of RR and OR indicated CC as risk factor genotype and CT as protective genotype for HCV-mediated HCC (Table 3).

**Table 3:** Genotype frequencies of CTLA4 + 49A/G and IL4 -590C/T polymorphism in HCV induced HCC patients and Control groups

Gene	Genetype	Patient (*n* = 213)	Control (*n* = 216	Odds ratio	95% CI –odds ratio	Relative risk	95% CI—relative risk	*P* value
		***n*, (%)**	***n*, (%)**					
CTLA4	AG	134 (62.9%)	55 (25.5%)	4.96	3.29 -7.52	2.154	1.76—2.64	< 0.0001
	GG	79 (37.1%)	161 (74.5%)	0.20	0.13—0.30	0.4643	0.37—0.56	< 0.0001
IL4	CC	138 (64.8%)	60 (27.8%)	4.78	3.19 -7.17	2.14	1.75—2.65	< 0.0001
	CT	46 (21.6%)	136 (62.9%)	0.16	0.11—0.25	0.37	0.28—0.48	< 0.0001
	TT	29 (13.6%)	20 (9.3%)	1.54	0.84—2.84	1.22	0.91—1.53	0.17

### Gender-based comparison of CTLA-4 + 49A/G and IL-4 -590 C/T genotypes in HCV-associated HCC patients

Gender based analysis of *CTLA-4* + 49A/G and *IL-4* -590 C/T genotypes revealed both AG and GG genotypes of *CTLA-4* were significantly associated with the disease. AG genotype in both genders was found as pathogenic while GG genotype was indicated as protective (Table 4). Similarly, genotypes CC and CT have significant association with HCV-induced HCC in both females and males. CC genotype in both genders eas indicated as risk factor while CT was shown to be protective (Table 4).

**Table 4:** Gender based genotype frequencies comparison of CTLA4 + 49A/G and IL4 -590C/T polymorphism in HCV induced HCC patients and Control groups

Gene	Gender based genotypes	HCV induced HCC %	Control %	Oddsratio	95% CI-odds ratio	Relativerisk	95% CI- relative risk	*P* value
		**Present**	**Present**					
CTLA4	Male AG	28.50%	18.65%	4.46	2.43—8.26	2.11	1.66–2.87	< 0.0001
	Male GG	18.65%	28.50%	0.22	0.12—0.41	0.47	0.34 -0.63	< 0.0001
	Female AG	33.62%	18.30%	5.32	3.06—9.35	2.16	1.66—2.85	< 0.0001
	Female GG	35.25%	64.75%	0.18	0.11—0.33	0.46	0.35 -0.60	< 0.0001
IL4	Male CC	32.64%	16.06%	5.15	2.80 -9.56	2.37	1.70—3.37	< 0.0001
	Male CT	9.84%	31.61%	0.17	0.09—0.34	0.37	0.24—0.55	< 0.0001
	Male TT	4.66%	5.18%	1.01	0.41—2.70	1.01	0.56 -1.51	> 0.99
	Female CC	31.91%	12.34%	4.62	2.68—8.04	2.01	1.56—2.62	< 0.0001
	Female CT	11.49%	31.91%	0.14	0.083 -0.25	0.37	0.26—0.51	< 0.0001
	Female TT	8.51%	3.83%	2.26	0.99—5.42	1.39	1.00—1.77	0.07

### Association of co-existence of CTLA-4 + 49A/G and IL-4 -590 C/T genotypes in HCV-associated HCC patients

In the present study, the influence of the co-existence of *CTLA-4* and *IL-4* genotypes in HCV-mediated HCC patients was also evaluated. Genotypes GG (*CTLA-4*) and TC (*IL-4*) co-existed in 11.74% of patients and demonstrated significant association with HCV-mediated HCC. However, OR and RR indicated their co-existence as protective. Likewise, genotypes CA (*CTLA-4*) and TT (*IL-4*) were found to have co-existence in 8.45% patients with significant association with disease. Contrary to genotypes GG (*CTLA-4*) and TC (*IL-4*), genotypes CA (*CTLA-4*) and TT (*IL-4*) were determined as risk factor. Similarly, genotypes GA (*CTLA-4*) and CC (*IL-4*) were also identified as risk factor and co-existed in around 45% of the patients (Table 5).

**Table 5:** CTLA-4 and IL-6 co-existing genotypes, along with their relative risk, odds ratio and p-value in HCV-induced HCC patients

Genotype (CTLA4 + IL6)	Number		Relative Risk (95% CI)	Odds Ratio (95% CI)	*P*-value
	**Patients (%)**	**Control (%)**			
GG + TT	11 (5.16%)	14 (6.48%)	0.88 (0.52—1.28)	0.78 (0.35—1.71)	0.68
GG + TC	25 (11.74%)	107 (48.61%)	0.31 (0.21—0.43)	0.140 (0.085—0.23)	< 0.0001
GG + CC	43 (20.19%)	40 (18.52%)	1.05 (0.82—1.31)	1.113 (0.68—1.81)	0.71
GA + TT	18 (8.45%)	6 (2.91%)	1.51 (1.10—1.85)	3.077 (1.26—7.56)	0.01
GA + TC	21 (9.86%)	29 (13.43%)	0.82 (0.57—1.12)	0.7053 (0.39—1.27)	0.29
GA + CC	95 (44.60%)	20 (9.26%)	2.19 (1.86—2.59)	7.890 (4.63—13.72)	< 0.0001

## Discussion

HCC incidence is decreasing in several high-risk countries, but its incidence is increasing in low-risk countries [33, 34]. There are many modified risk factors linked with HCC that increase association of an individual with HCC, such as alcohol intake, smoking or toxin exposure. Apart from extrinsic factors, genetic polymorphism is also identified as a major contributor. HCV infection is among the prominent causes of HCC [35]. The present study aimed to identify whether *CTLA-4* + 49 A/G and *IL-4*–590 C > T polymorphisms associate in HCV-induced HCC Pakistani patients. A further aim was to evaluate potential pathogenic contributions of these polymorphisms to HCC.

The *IL-4* and *CTLA-4* SNPs were localized to the promoter region and protein-coding region, respectively. RegulomeDB analysis indicated likely regulatory roles and transcription factor binding sites for the *IL-4* SNP, whereas gene regulatory roles of the *CTLA-4* polymorphism were estimated as low. The *CTLA-4* SNP represents as missense variant, and pathogenicity of this SNP was estimated through in silico SNP analysis tools: SIFT, PolyPhen2.0, CADD, REVEL, metaLR and MutationAssessor. Scores from each tool classified both *CTLA-4* + 49 A/G and *IL-4*–590 C/T SNPs in tolerant or benign class.

Evidence from literature indicated the involvement of the *CTLA-4* + 49 A/G variant in a number of diseases such as rheumatoid arthritis [36], Behcet’s disease [37], Graves’ disease [38], diabetes mellitus, thyroid diseases and colorectal cancer [39]. However, information is lacking on the relationship of the *CTLA-4* + 49 A/G polymorphism and HCV induced HCC in the Pakistani population. In a study conducted among Chinese population, *CTLA-4* + 49 A/G association with HBV induced HCC was concluded [40]. *CTLA-4* polymorphism rs3087243 G > A also showed association with HBV induced HCC among eastern Chinese Han population [17]. *CTLA-4* polymorphism along with IL-10 and TNF-alpha also reported associated with HCC [23]. In the present study, *CTLA-4* + 49 A/G polymorphism was identified as a potential risk factor for HCV induced HCC in Pakistan. Genotype AG was found to be in high frequency in HCV induced HCC patients while the dominant genotype in control samples was GG. The role of genotype GG was found as protective in HCV-induced HCC patient.

Allele G in *CTLA-4* + 49 causes threonine replacement with alanine. Molecular docking of *CTLA-4* wildtype and mutated protein with Sorafenib and Avastin indicated that alanine substitution caused an additional covalent bond between *CTLA-4* and the drugs. Formation of a covalent bond between ligand and protein is irreversible and a very strong interaction [41]. In most natural biological systems non-covalent interactions are typical [42], and these interactions in comparison to covalent bonding are weak and reversible [41, 42]. The presence of a covalent bond between mutated *CTLA-4* protein and Avastin and Sorafenib dictates a strong interaction. This further hints towards the protective influence of *CTLA-4* GG genotype that might enhance therapy influence.

Studies conducted earlier provided insight into the possible role of *IL-4* polymorphism with HCV-induced HCC. The outcomes of the current study pointed towards the association of -590 C > T mutation of *IL-4* with increased risk of HCV-induced HCC, and align with a previous study including *IL-4* [43]. Annotation scores from RegulomeDB for -590 SNP indicated the regulatory role of this SNP and the presence of transcription factor binding sites. This finding was further confirmed by Alibaba 2.0 analysis. The C > T mutation created 2 additional binding sites, MIG1 and SP1. SP1 is located within the E1 region that is responsible for activity of enhancers in *IL-4*. Such SP1 mutation was thought to have no effect on normal functioning of the E1 region, but more research is needed to draw any conclusion [44]. These mutations can alter the normal cellular functions, which may lead to cancer formation. These findings support -590 C > T (rs2243250) involvement in hepatocellular carcinoma.

*IL-4* -590 C > T polymorphism has a direct association with cancers [24], and is linked with certain diseases such as atopic asthma & allergic rhinitis [25] and smoking linked cancer [36]; however, many studies report that this polymorphism does not have a significant association with disease incidence [45]. A study linked with the Egyptian population showed that *IL-4* -590 C > T polymorphism was associated with HCV induced HCC. *IL-4* can promote macrophage and Th cell production, which in turn supports progression of cancers like HCC [43]. *IL-4* -590 C > T polymorphism is associated with both HBV and HCV induced HCC in a Caucasian population [46]. In our study cohort, genotype “CC” was found to be in high frequency in HCV induced HCC patients and is indicated as risk factor allele.

## Conclusion

In the present study, *CTLA-4* + 49 genotype AG and *IL-4* -590 genotype CC were found to be associated with the pathogenicity of HCV-mediated HCC. Age-based and gender-based analysis revealed the association of *CTLA-4* AG genotype with adults (age 20–39) and both genders. Similarly, *IL-4* association with age groups 20-39yrs and 40-59yrs and with both genders was revealed. Computation analysis estimated that *CTLA-4* polymorphism might have influence on the hydrophobicity and structure of the protein while *IL-4* polymorphism might lead to altered transcription factor binding sites in its promoter region. The *CTLA-4* polymorphism might augment efficacy of clinically available chemotherapy drugs. Thus *CTLA-4* & *IL-4* SNP polymorphisms have strong association with HCV induced HCC among Pakistani patients that may have application to serve as genetic markers for pre- and early diagnosis and prognosis of HCC in HCV patients.

## Data Availability

All data generated or analyzed during this study are included in this article.
